# An Evaluation of the Usability of ReACT (Responsive Asthma Care for Teens), an Adaptive Mobile Health Intervention for Adolescents With Asthma: Feasibility and Acceptability Trial

**DOI:** 10.2196/63905

**Published:** 2026-05-26

**Authors:** Christopher C Cushing, Calissa Leslie-Miller, Natalie Koskella-Staples, Adrian Ortega, Helenna Shcherbinin, Shreekala Prabhakaran, David A Fedele

**Affiliations:** 1 Department of Clinical Child Psychology, Life Span Intitute, University of Kansas, 1000 Sunnyside, Lawrence, KS, 66045, United States, 1 4172250629; 2 Pediatric Pulmonary Division, University of Florida, Gainesville, FL, United States; 3 Developmental and Behavioral Health, Northwestern University, Chicago, IL, United States; 4 Center for Healthcare Delivery Science, Nemours Children's Health, Jacksonville, FL, United States

**Keywords:** mHealth, asthma, feasibility, usability, Responsive Asthma Care for Teens, ReACT

## Abstract

**Background:**

Adolescent asthma is a significant contributor to youth morbidity and is known to be best managed through consistent medication use and symptom management. However, adolescents often struggle to perceive their symptoms accurately and consistently use their medication at the recommended rate, risking worsened symptoms and impaired quality of life. The Responsive Asthma Care for Teens (ReACT) system is a project aimed at identifying and and providing supporting for several barriers adolescents may face in asthma management. By integrating both software and hardware to monitor medication adherence, ReACT provides a personalized support plan to improve asthma management and, subsequently, quality of life.

**Objective:**

The objective of this study is to conduct a proof-of-concept assessment of the ReACT system following an initial pilot study and adjusting for the feedback received. In addition to assessing the acceptability and usability of the current version, this study aims to assess whether the proposed ReACT system leads to indications of improvement in medication adherence because of the personalized support plans.

**Methods:**

Participants in the study were 5 adolescents aged 15 to 17 years recruited using a combination of consent-to-contact forms delivered via an in-person asthma clinic and Qualtrics panels. As a part of this study, participants met with the study team 3 times over 1 month. After completing initial surveys on stress, problem-solving, and asthma-related quality of life, we oriented the participants to the ReACT platform and asked them to interact with it as normal. After the month, the participants were interviewed, and they discussed the system and completed surveys assessing their opinions on acceptability and usability.

**Results:**

On a 4-point scale, participants reported high acceptability of ReACT (mean score 3, SD 0.32), willingness to use it again (mean score 4, SD 0.89), and willingness to recommend it to a friend (mean score 3.75, SD 0.55), and they considered it to be helpful (mean score 3.2, SD 0.84).

**Conclusions:**

Our findings suggest that ReACT is an acceptable and usable mobile health intervention to improve asthma self-management among adolescents, and it had promising results for improving self-regulation, problem-solving, and asthma control. The system continues improving based on feedback from a larger sample size of participants, and we hope that ReACT will aid adolescent development while delivering highly personalized support for each user.

## Introduction

Pediatric asthma affects 9% of American adolescents [1]. Asthma is a leading cause of youth morbidity [2,3] and impaired quality of life. For youth with persistent asthma, adherence to effective self-management behaviors, such as using inhaled corticosteroids (ICSs) and monitoring symptoms, is key for controlling daily asthma symptoms and preserving long-term lung health [4]. Adolescents with asthma frequently have difficulties with consistent engagement in self-management behaviors. Adolescents use ICSs less than half the recommended time according to electronic monitoring data and often struggle with accurate symptom perception [5-8]. Suboptimal self-management elevates adolescents’ risk of worsened asthma control, heightens morbidity, such as symptom flares and emergency department visits, and diminishes quality of life [5,9].

The reasons for asthma self-management difficulties during adolescence are multifactorial. Adolescence presents unique challenges to self-management due to factors such as academic responsibilities, managing social relationships, future planning [10-12], underdeveloped self-regulatory abilities (cognitive, emotional, and behavioral) [13,14], and a growing desire for autonomy from caregivers [14-16]. Our research team has highlighted that these developmental transitions often align with an increase in dynamic stressors that negatively affect self-management [17].

In response to these challenges, our team developed the Responsive Asthma Care for Teens (ReACT) system, a project using the model from the Obesity-Related Behavioral Intervention Trials (ORBIT) consortium for designing and evaluating behavioral interventions [18]. ReACT is an adaptive, personal self-management support system comprising a network of interconnected IT components that can be tailored to individual users’ needs. The ReACT system integrates both hardware and software elements that passively monitor a participant’s adherence to ICSs and subsequently provide personalized goal-setting, feedback, barrier identification, and problem-solving training. By bolstering self-regulation, problem-solving skills, and medication adherence, ReACT intends to enhance asthma control and asthma-related quality of life. We recently completed phase 1 of the ORBIT model by designing ReACT in collaboration with adolescents with asthma (n=280) through iterative design sessions, during which they provided feedback on key system features and content [19,20].

The main goal of the current project was to conduct a proof-of-concept assessment of ReACT (ORBIT phase 2a) by evaluating its acceptability and usability in advance of a larger intervention trial (a transition toward phase 2b—early efficacy testing). Secondarily, we aimed to assess the ability of ReACT to engage our hypothesized putative mechanisms of action, namely problem-solving skills and self-regulation abilities. We anticipated that participants would express satisfaction with the ReACT platform, they they would effectively use the system, and that there would be initial evidence that ReACT could engage our proposed mechanisms of action for a larger-scale trial, moving forward in the ORBIT model progression.

## Methods

### Recruitment

We recruited 5 participants for the initial usability test of ReACT. Participants were adolescents aged 15 to 17 years (1 boy and 4 girls) with a current asthma diagnosis requiring regular ICS use for at least 6 months. Participants were included if they a daily ICS prescription that could accommodate the ReACT sensor (ie, mometasone/formoterol or fluticasone), were able to read and speak in English, and had their own smartphone. Adolescents were excluded if they were currently involved in another asthma management intervention, had a comorbid health condition that could impact lung function (eg, cystic fibrosis), or had significant cognitive impairments or developmental delays that would interfere with study completion.

### Procedures

We recruited participants using a combination of consent-to-contact forms delivered via an in-person asthma clinic (University of Florida) and a national crowdsourcing campaign seeking to identify youth with asthma via a brief survey through Qualtrics Panels.

### Ethical Considerations

All study procedures were reviewed and approved by the Institutional Review Board at the University of Kansas Medical Center and the University of Florida (STUDY00144292). Informed consent was collected from all legal guardians, and assent was collected from all participants. Data were deidentified and stored on secure servers behind a university firewall. Participants were compensated up to US $175 for completing all 3 study meetings.

### ReACT Platform

ReACT is an adaptive, personal self-management support system consisting of educational modules delivered via animated videos, a mobile sensor, and a smart hub for assessing medication adherence, as well as a cloud-based platform managed by Way to Health that houses the intervention rules engine and triggers SMS messages to be delivered to participants based on their adherence. When users are onboarded to ReACT, they are given access to videos that provide asthma education, a basic understanding of problem-solving training, and an overview of ReACT features. The ReACT smart hub was configured to continuously scan for the ReACT sensor and transmit adherence data to the cloud. Once adherence data were received in the cloud, the ReACT rules engine was used to determine what intervention content should be delivered to the participant. ReACT monitors several aspects of adherence data, including whether rates are increasing, decreasing, or stable (over a 3-day window); whether rates are highly variable, or if extremely high or low values (ie, < or >50% adherence) are recorded. Depending on the combination of these factors, participants received different intervention content. If adherence declined for 6 consecutive days, then ReACT would assess barriers to adherence and walk participants through an automated, personalized problem-solving intervention via text [19,20].

### Study Visits

Participants completed 3 study visits over the course of 1 month. At the first visit, participants provided informed consent and assent and completed self-report questionnaires assessing study mechanisms via REDCap. After the first visit, participants were given instructions on how to attach the ReACT sensor to their ICS inhaler and ensure a data connection with the ReACT smart hub by keeping it plugged into a standard wall outlet. Participants were then shown the 3 introductory videos for the ReACT platform and instructed on how to interact with ReACT over the next 4 weeks. At the end of the visit, participants conducted a “test out” procedure to ensure understanding, and we conducted a troubleshooting call to ensure data connectivity after approximately 3 days for each participant. At the end of 4 weeks, participants repeated the self-report assessment of study mechanisms and completed measures assessing satisfaction and usability before a one-on-one semistructured interview that assessed satisfaction, usability, and suggestions for improvement of ReACT.

### Qualitative Coding

Two researchers (NK-S and CL-M) independently read transcripts of the one-on-one semistructured interviews. The researchers identified important concepts and created a codebook guided by self-regulation theory, although de novo themes also emerged from interviews. The researchers coded the first 2 transcripts independently and then met to resolve discrepancies and refine the codebook. Transcripts were coded for (1) self-regulation theory components (ie, goal setting, problem-solving, and thinking about management), (2) medication adherence and control, (3) components participants enjoyed, (4) barriers to regular use, or (5) modifications. The researchers independently coded the remaining 3 transcripts and discussed all discrepancies until reaching consensus.

### Measures

#### Client Satisfaction

The 8-item Client Satisfaction Questionnaire (CSQ-8) [21]was used to assess participants’ satisfaction with ReACT (Cronbach α=0.90). Participants were asked to answer 8 items on a 4-point response scale.

#### Usability

The System Usability Scale (SUS) [22] was used to assess participant perceptions of the usability of ReACT. Participants were asked to indicate their level of agreement with each of the 10 items on a 5-point Likert scale (Cronbach α=0.74).

## Results

### Acceptability and Usability

Table 1 shows the demographics of the participants. Participants reported high satisfaction with ReACT (mean score 3, SD 0.32 on a 4-point scale) on the CSQ-8. Adolescents said they would use ReACT again (mean score 4, SD 0.89), recommend it to a friend (mean score 3.75, SD 0.55), and that ReACT was helpful (mean score 3.5, SD 0.84). Adolescents rated ReACT as more usable than 70% of computerized systems on the SUS, which is above the threshold for acceptability based on normative data [22].

**Table 1.** Participant demographic data (N=5).

Characteristics		Values
Age (years), mean (SD)		16.20 (0.84)
Age (years), n (%)		
	15	1 (20)
	16	2 (40)
	17	2 (40)
Gender, n (%)		
	Male	1 (20)
	Female	4 (80)
Race/ethnicity, n (%)		
	White/Hispanic	1 (20)
	White/non-Hispanic	1 (20)
	Black or African American	3 (60)

Table 2 shows themes for ReACT self-regulatory processes, adherence outcomes, and feedback endorsed by participants. Participants indicated that they “really enjoyed” ReACT and found it “very organized.” Overall, the youth indicated many components that they enjoyed in ReACT. The messages were presented as the primary preferred content compared to the videos. Participants liked the messages because they “[make] you want to get up and go take your medicine, because it shows you how many doses you’ve missed exactly.” This personalization was heavily regarded as beneficial and was requested in more components of the application, such as the color of the equipment and the timing of messages.

**Table 2.** Youth-endorsed self-regulatory processes, adherence outcomes, and feedback.

Interview theme	Participants who endorsed, n (%)	Number of times mentioned	Description of theme
Self regulation theory	5 (100)	36	Youth mention goal setting, problem-solving, or thinking about management.
Adherence control	5 (100)	22	Youth mention taking medication.
Enjoy	5 (100)	50	Youth describe a component of ReACT that they enjoyed.
Barrier to regular use	5 (100)	31	Youth explain a component of ReACT that would inhibit regular use.
Change/add	5 (100)	70	Youth suggest a modification.

The participants provided a variety of suggestions for modifications, which included more variability in the messages, as they were perceived as “dull.”

Yeah, it got kind of old, because it was just like yes, yes, yes.

I liked it, but it could be more appealing to the audience it’s meant for. It was just dull.

Dull messages with yes or no questions on them, so I guess where it could improve a little bit more, like have better phrases of questions and stuff like that.

Participants were interested in a more adaptive and personalized intervention experience, requesting “options to talk back to you” or “a bank of messages and it’s just randomized and sent out, so it’s like you have to pay attention to it to actually respond to it.” An additional suggestion for modifications included reminder messages “if it didn’t get a response within a certain period of time.” Participants also indicated a desire for a “hands-on tool teaching you how to take your medicine.” Additionally, there were concerns regarding technical challenges, specifically that completed doses were not being processed as completed. Participants indicated that the sensor on the inhaler “was kind of buggy, because it worked sometimes and then it didn’t work sometimes.”

The text messages were pretty long. That’s about it for the text messages. The sensor was not working, so that was it for that.

### Evidence of Changes in Outcomes

Findings from qualitative interviews revealed near consensus that ReACT increased medication adherence and control, and this was mentioned a fair number of times. Consistently, participants indicated that ReACT helped in remembering to take medication: “They send off a text message, and I remember, so they help me a lot to take my medicine.” There were indications of notable changes because of ReACT:

I never really took my meds until I got into this program.

It made me realize that I go through periods of taking my medication, or if I don’t feel like it, I won’t… And so, I didn’t realize the consistency of how I took my medication, which I now take quite consistently.

It helped me pretty good, because sometimes I would forget to take [my medication], and sometimes, even if I was feeling a little down, it would remind me to get it. It would basically encourage me, tell me how important my breathing really is, because it only takes one situation to happen and you can have a very bad asthma attack. So, it definitely helps you remember to take your stuff on time.

Interview responses suggested that problem-solving, self-regulation, motivation, and stress-management components assisted in increasing medication adherence. For example, “I set a goal of taking it, and I completed it. I made a goal, and I stuck to it, so I think ReACT played a part in that.” ReACT messages successfully reminded participants about asthma and medication goals. Another participant indicated that the steps integrated into the agenda helped problem-solve their barriers to medication adherence. Additionally, personalized reminders via text messages were successful and helped the participants remember to take their medicine. For example, one participant noted that ReACT helped them “remember that I had a goal that I should take the medication, because it was on my phone and it was in front of me.” Another stated that “it makes you want to take it more. It was like, you only took two doses of your medicine this whole week. I was like, Oh, that’s not good, and so it helps a lot when it shows you how many doses you were missing.” Themes from exit interviews revealed that the participants were satisfied with ReACT and that they improved their regulatory abilities, could better monitor their symptoms, and adhered to their regimen because of ReACT.

## Discussion

### Principal Results

This proof-of-concept study established that ReACT is highly usable and acceptable to adolescents with chronic and persistent asthma, justifying additional investigation. Participants were engaged by hypothesized intervention features targeting self-regulation and barriers to adherence. Consistent with our first aim, participants exceeded our satisfaction target on the CSQ-8 and gave above-threshold scores on usability (SUS). In support of our second aim, the qualitative data revealed that ReACT may engage the putative mechanisms of action, such as goal-setting, self-monitoring of adherence, and problem-solving.

Our findings generate confidence that ReACT shows promise as an acceptable and usable mobile health (mHealth) intervention to improve asthma self-management among adolescents. Regarding quantitative data, the participants’ ratings of ReACT exceeded well-defined a priori cutoffs for acceptability (score ≥2.5 on a 4-point scale) and usability (scores ≥2 on a 5-point scale) [21,22]. Several themes from our qualitative data corroborated the quantitative usability and acceptability ratings. Participants reported general enjoyment with ReACT and found the text messages to be informative regarding their adherence to ICS use. Moreover, participants responded positively to how ReACT felt personalized and timely based on their unique patterns of ICS use.

An important goal of the current study was to identify ways to modify ReACT from the perspective of target users in advance of transitioning toward efficacy testing. Indeed, it is a typical and expected part of the ORBIT process that interventions undergo additional refinement after pilot-testing and before a randomized controlled trial [18]. Our project revealed opportunities to improve ReACT, such as the comments that large text interactions with a series of yes/no questions are unpleasant and should be revised. Furthermore, the participants indicated that they would welcome additional opportunities for more personalization of intervention content and, if needed, engagement via text message with study staff. Users also noted concerns that some of the intervention features did not perform as intended, which should be addressed before moving into a clinical trial phase.

### Limitations

The current project is a small pilot study of a small number of patients who were homogeneous concerning age and medication regimen. Moreover, the current project occurred before recommendations for single maintenance and reliever therapy were part of the pediatric guidelines. ReACT will need to be reconfigured to apply broadly to the newest regimens for patients with asthma.

### Comparison With Prior Work

These results are consistent with previous literature. The high acceptability and usability of ReACT is consistent with a recent systematic review of digital interventions in pediatric asthma, which concluded that adolescents with asthma find mHealth interventions to be a viable mechanism to promote self-management [23]. Additionally, there is burgeoning literature demonstrating that mHealth interventions that deliver highly personalized and timely intervention content to users during times of need may be perceived as helpful in changing targeted health behaviors. Thus, the ability of ReACT to generate adaptive and personalized self-management promotion messages for adolescents with asthma, especially during times of acute need (eg, declining adherence to ICS use), may be particularly helpful compared to many existing mHealth solutions for this population that rely on static content and reminder alarms.

### Conclusions

Collectively, these positive and theoretically consistent findings suggest that ReACT promises to improve self-regulation, problem-solving, and asthma control. Interventions targeting asthma self-management have been stagnant for some time [6]. One hypothesis for how to break through this stagnation is to provide more personalized support that fills the critical self-regulatory gaps common to adolescent development.

## Abbreviations

CSQ-8: 8-item Client Satisfaction Questionnaire
ICS: inhaled corticosteroid
mHealth: mobile health
ORBIT: Obesity-Related Behavioral Intervention Trials
ReACT: Responsive Asthma Care for Teens
SUS: System Usability Scale
